# Well-leg compartment syndrome after robot assisted laparoscopic surgery for rectal cancer: A case report

**DOI:** 10.1016/j.ijscr.2023.107924

**Published:** 2023-02-14

**Authors:** Keiichi Arakawa, Akihiro Sako

**Affiliations:** Department of Surgery, Hitachi General Hospital, Japan

**Keywords:** Well leg compartment syndrome, Robot, Rectal cancer

## Abstract

**Introduction:**

Lower limb compartment syndrome caused by improper positioning during surgery is called well-leg compartment syndrome. Although well-leg compartment syndrome has been reported in urological and gynecological patients, there have been no reports of well-leg compartment syndrome in patients who have undergone robot-assisted surgery for rectal cancer.

**Presentation of case:**

A 51-year-old man was diagnosed with lower limb compartment syndrome by an orthopedic surgeon due to pain in both of his lower legs immediately following robot-assisted surgery for rectal cancer. Due to this, we started placing the patient in the supine position during these surgeries, and repositioned the patient to the lithotomy position following intestinal tract cleansing after rectal movement in the latter half of the surgery. This avoided the long-term effects of being in the lithotomy position. We compared the operation time and complications before and after the above measures were changed, in 40 cases of robot-assisted anterior rectal resection for rectal cancer performed at our hospital from 2019 to 2022. We found no extension of operation time and no occurrence of lower limb compartment syndrome.

**Discussion:**

There have been several reports describing the risk reduction of WLCS using intraoperative postural changes. An intraoperative postural change from a natural supine position without pressure which we reported is considered to be a simple preventive method for WLCS.

**Conclusion:**

Changing the patient from the supine position to the lithotomy position during surgery may be a clinically acceptable countermeasure to prevent lower limb compartment syndrome.

## Highlights

• A man who was diagnosed with leg compartment syndrome after robotic rectal cancer surgery
• Changing the patient from the supine position to the lithotomy position during surgery
• Short term outcome in 40 cases of robot-assisted anterior rectal resection for rectal cancer

## 1 Introduction

Compartment syndrome is often traumatic, but can occur non-traumatically. Lower limb compartment syndrome due to improper positioning of the lower limbs during surgery is referred to as well-leg compartment syndrome (WLCS) [1]. WLCS was first described by Leff et al. when it developed in a patient in the lithotomy position during a total cystectomy [1]. The majority of reports on WLCS are associated with lithotomy surgery, with Halliwill et al. defining the incidence as 1 in 3500 cases [2]. There has been an increase in robot-assisted surgeries in recent years. WLCS is a complication that should be dreaded even in robot-assisted laparoscopic surgery (RALS), such as RALS for rectal cancer performed in the lithotomy position [3]. To date, 22 cases of WLCS following laparoscopic surgery for rectal cancer, but no cases of WLCS following RALS for rectal cancer have been reported [4]. Here, we describe WLCS in a patient that occurred during RALS for rectal cancer. We describe the measures taken, along with reporting the short-term outcome in the patient treated in our hospital. This paper was written in compliance with the SCARE 2020 Guideline [5].

## 2 Presentation of case

**Fig. 1:**
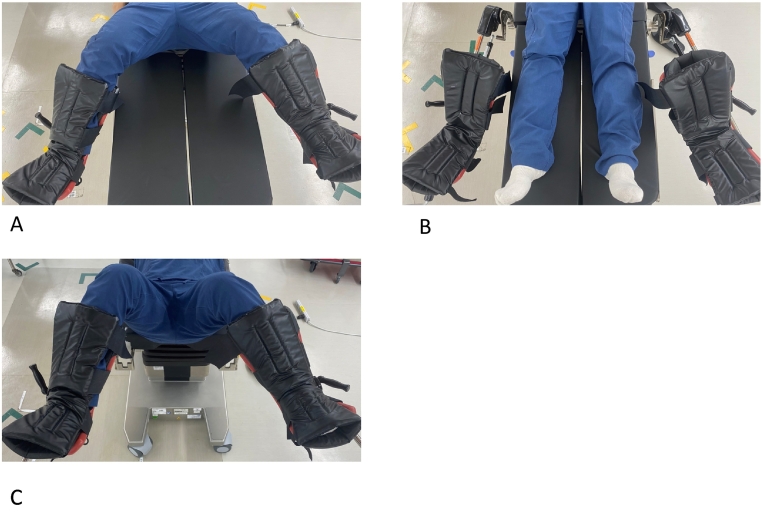
A. Confirm the lithotomy position before surgery. B. Start surgery at supine position. C. Change the patient from supine to lithotomy after transanal irrigation.

**Fig. 2:**
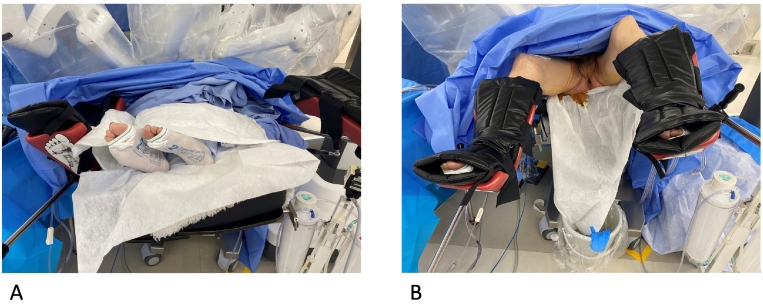
A. Beginning of the surgery when the robot rolls in and the patient is in the supine position. B. The patient is changed from the supine to lithotomy position during the second half of the surgery as the robot is rolling in.

A 51-year-old man with a body mass index (BMI) of 25.9 was referred to our hospital with the chief complaint of melena. RALS for rectal cancer was performed under general anesthesia, with the patient in the lithotomy position. After induction of anesthesia, a leg holder was used to set the patient in the lithotomy position before surgery, and a rotation test was performed with the patient in the same head down position (12° head down position) and right rotation (12°) as used during surgery. This confirmed that there is no displacement of the body. Elastic stockings were used to prevent thrombosis, and an intermittent pneumatic compression device was installed to pressurize the lower leg. The operating time was 6 h and 24 min, and blood loss was 100 ml. Pain in both lower legs was recognized at the time of awakening from anesthesia immediately after surgery. Ultrasonography of the lower extremities revealed a 4 × 2 cm hematoma inside the left peroneus muscle and a small hematoma inside the right peroneus muscle. The patient was diagnosed with lower extremity compartment syndrome. Since there was no neuropathy or blood flow disorder beyond the lower leg, we decided to treat conservatively with leg elevation and cooling. At 12 days post-surgery, the patient was discharged home without neurological sequelae in the lower leg. Lower extremity compartment syndrome in this case was caused by the prolonged time the patient had their head down and was in the lithotomy position. Subsequent to this case, our hospital took countermeasures when using RALS for rectal cancer. In our amended procedure, the leg holder used to place the patient in the lithotomy position is first mounted on the operating table before the start of surgery. Once both legs are placed on the leg holder to confirm the patient is in the lithotomy position, the patient is returned to the supine position and the surgery is started (Fig. 1A). This also facilitates the transition from the supine position to the lithotomy position during surgery. Therefore, the patient should be in the supine position at the start of the surgery, and the head down position should be added as the surgery progresses (Figs. 1B, 2A). Next, after transanal irrigation is completed following the mesorectal treatment in the second half of the surgery, the surgical operation is stopped for a short time while the arm of the robot remains docked. Placing both legs on the leg holder that was set in advance possibly reduces the time the patient is in the lithotomy position (Figs. 1C, 2B). We compared the short outcomes of patients who underwent RALS for rectal cancer in our hospital before and after we made the amendment to our procedure. The overall characteristics compared before and after introduction of the countermeasures are displayed in Table 1. There were no significant differences in any of the characteristics, such as operation time or complications, before and after the countermeasures were employed. Further, no further cases of WLCS due to RALS have been observed since the aforementioned case.

**Table 1:** Clinicopathological features of 40 cases of RALS for rectal cancer.

		Total cases		Before		After		p value
		N = 40		N = 17	(42.5 %)	N = 23	(57.5 %)	
General variables								
Age (years)	Median	69		69		67		0.8380
	Range	47–86		47–86		52–79		
Sex, N (%)	Male	33	(82.5)	15	(88.2)	18	(78.3)	0.6770
	Female	7	(17.5)	2	(11.8)	5	(21.7)	
BMI, N (%)	Median	22.1		21.6		22.1		0.5680
	Range	17.9–27		18.1–27		17.9–26.6		
Operative time (min)	Median	282		290		277		0.4123
	Range	181–428		202–428		181–397		
Inferior mesocolic artery	High tie	16	(40.0)	8	(47.1)	8	(34.8)	0.5219
	Low tie	24	(60.0)	9	(52.9)	15	(65.2)	
Hospital day	Median	12		12		12		0.4995
	Range	9–40		10–22		9–40		
Clavien-Dindo	0–1	37	(92.5)	15	(88.2)	22	(95.7)	0.5647
	2–	3	(7.5)	2	(11.8)	1	(4.3)	
Pathological variables								
Tumor location	RS	16	(40)	7	(41.2)	9	(39.3)	0.1802
	Ra	20	(50)	10	(58.8)	10	(43.5)	
	Rb	4	(10)	0	(0)	4	(17.4)	
T stage	0	1	(2.5)	1	(5.8)	0	(0)	0.2795
	1	7	(17.5)	1	(5.8)	6	(26.1)	
	2	11	(27.5)	6	(35.3)	5	(21.7)	
	3	20	(50.0)	9	(52.9)	11	(47.8)	
	4	1	(2.5)	0	(0)	1	(4.4)	
Lymph node metastasis	Absent	25	(62.5)	10	(58.8)	15	(65.2)	0.7486
	Present	15	(37.5)	7	(41.2)	8	(34.8)	
Other organ metastasis	Absent	39	(97.5)	17	(100)	22	(95.7)	0.3839
	Present	1	(2.5)	0	(0)	1	(4.3)	
Stage	0	1	(2.5)	1	(5.9)	0	(0)	0.2798
	I	13	(32.5)	3	(17.7)	10	(43.5)	
	II	11	(27.5)	6	(35.3)	5	(21.7)	
	III	14	(35.0)	7	(41.2)	7	(30.4)	
	IV	1	(2.5)	0	(0)	1	(4.4)	

BMI: body mass index.

## 3 Discussion

Here, we describe a case of WLCS in a patient treated with RALS for rectal cancer. We report that we achieved a favorable short outcome by reducing the risk of WLCS by performing intraoperative postural changes of the patient.

The pathogenesis of WLCS is attributed to external pressure and peripheral circulatory failure [6]. Regarding the intraoperative position, Turnbull et al. reported that intramuscular pressure at the dorsal side of the lower leg in the leg holder is increased in the lithotomy position compared to the supine position [7]. They measured the pressure in the lower leg compartment during surgery and reported that body position affects the pressure in the lower leg compartment, which supports our present report [7]. When ischemic damage to the crural muscles occurs due to increased leg pressure, cell edema formation and cell swelling begin, and venous return damage progresses, further increasing compartment internal pressure, impairing arterial return, and increasing serum creatine kinase levels [8], [9]. Symptoms associated with blood flow disturbances are leg pain, swelling, pain induced by passive movement, and paresthesia. The clinical diagnosis of WLCS is made when these symptoms are included [10]. In addition to intraoperative positioning, risk factors for WLCS include prolonged surgery duration, history of peripheral vascular disease, use of elastic stockings and intermittent pneumatic compression devices, intraoperative peripheral circulatory failure, and traction and compression of the lower extremities and blood vessels [11]. In addition, it is considered that there is a correlation between BMI and intra-compartment pressure, and that the weight of the leg reduces the volume of the compartment and increases the intra-compartment pressure [12]. In our case, in addition to the patient being in the head down and lithotomy position, the operating time of more than 6 h, the use of intermittent pneumatic compression, and a BMI of more than 25 were considered risk factors.

Treatment for WLCS includes removal of pressure factors and relaxing dissection. Although there is no consensus regarding the indications and timing of relaxing incision, many reports suggest that the incision should be performed when the compartment pressure is 30–55 mm Hg or higher [13], [14]. In our case, the patient's symptoms were mild. Based on the diagnosis by an orthopedic surgeon using lower extremity ultrasonography, conservative treatment with rest and cooling resulted in improvement of symptoms.

WLCS tends to be detected late due to the effects of anesthesia, and many reports describe a poor prognosis compared to general compartment syndrome caused by trauma. A summary of 65 WLCS cases reported that 4 patients died and 11 had amputation [15]. In recent years, rectal cancer surgery has often been performed under laparoscopic or robotic assistance. Laparoscopic and robotic surgeries have been pointed out as key factors in the development of WLCS [4], [16]. Although there have been no reports of WLCS due to RALS for rectal cancer, the frequency of WLCS in robot-assisted radical prostatectomy in the field of urology is 0.29 %, which is higher than 0.03 % in open surgery [2], [17], [18]. Although the surgical method for RALS for rectal cancer is different from that of urology, it involves robotic surgery with the patient in the lithotomy position. There is concern that cases of WLCS will increase in the future, even for RALS for rectal cancer, which is becoming more prevalent. Considering this, gastroenterological surgeons should be more thorough in preventing WLCS because, although rare, it can cause serious sequelae if it develops. Although there are currently no established preventive measures for WLCS, avoidance of lithotomy position may improve intracompartmental pressure and peripheral circulatory insufficiency, preventing WLCS [2], [19]. There have been several reports describing the risk reduction of WLCS using intraoperative postural changes, such as temporarily canceling the lithotomy position 4 h into surgery and using an open leg position for procedures other than those requiring a lithotomy position [19], [20]. An intraoperative postural change from a natural supine position without pressure, which we reported as a preventive measure for WLCS in RALS, is considered to be a simple preventive method for WLCS that can be safely performed at any facility without requiring special techniques or materials. As we encountered a case of WLCS during robot-assisted rectal resection for rectal cancer, we devised a method to suppress the onset of WLCS by changing the patient's position from the supine position to the lithotomy position during surgery.

## Consent for publication

Written informed consent was obtained from the patient for the publication of this case report and any accompanying images. A copy of the written consent is available for review by the Editor-in-Chief of this journal.

## Ethical approval

The requirement for institutional review board approval was waived because this case report did not contain content required for ethical approval.

## Funding

N/A.

## Guarantor

Corresponding author; Keiichi Arakawa.

## Research registration number

N/A.

## CRediT authorship contribution statement

All authors were both involved in the conception and coordination of this report and drafted the manuscript. Additionally, both authors have read and approved the final version.

## Conflicts of interest

N/A.

## Data Availability

All data on which the conclusions of this case report are based are included in this manuscript.
